# Primary extraskeletal osteosarcoma of the esophagus with PPM1D-BCAS3 fusion: a case report and literature review

**DOI:** 10.3389/fmed.2025.1558831

**Published:** 2025-06-25

**Authors:** Shuai Luo, Xiaoxue Tian, Ting Xu, Qing Ji, Yao Li, Jinjing Wang

**Affiliations:** Department of Pathology, Affiliated Hospital of Zunyi Medical University, Zunyi, Guizhou, China

**Keywords:** extraskeletal osteosarcoma, esophageal, pathological diagnosis, differential diagnosis, gene fusion

## Abstract

**Background:**

Extraskeletal osteosarcoma (ESOS), a malignant soft tissue neoplasm characterized by the presence of osteoid or cartilage matrix, arises without bone involvement. ESOS within the esophagus is particularly rare. This study reported a case of esophageal ESOS with PPM1D-BCAS3 fusion, offering an analysis of its clinicopathological features and a comprehensive literature review aimed at advancing the diagnostic understanding of clinical pathologists.

**Case demonstration:**

A 76-year-old male presented with a 2-month history of subxiphoid pain and dysphagia. Chest computed tomography CT revealed irregular mid-esophageal wall thickening and luminal stenosis, raising suspicion for mid-esophageal carcinoma. Painless endoscopic ultrasonography detected a hypoechoic intraluminal esophageal mass. Endoscopic submucosal dissection (ESD) and histopathological analysis confirmed primary ESOS of the mid-esophagus. No adjuvant therapy was administered postoperatively. However, the patient experienced a recurrence with multiple metastases 9-months later.

**Conclusion:**

The diagnosis of esophageal ESOS, a condition of exceptional rarity, necessitates an integrative evaluation that encompasses clinical history, imaging modalities, and histopathological analysis, with a critical distinction from metastatic osteosarcoma and carcinosarcoma. This study presented the first case of esophageal ESOS with PPM1D-BCAS3 fusion treated via ESD, providing a significant point of reference for clinicians and pathologists. The management of ESOS demands vigilant monitoring and precise follow-up care.

## Background

Extraskeletal osteosarcoma (ESOS), a malignant neoplasm of mesenchymal origin, was first identified by Wilson in 1941 (1). Representing merely 1–2% of all soft tissue sarcomas and 2–4% of osteosarcomas (2, 3), ESOS predominantly affects elderly males, manifesting primarily as soft tissue masses in the limbs, particularly in the thighs (lower limbs, 46.6%; upper limbs, 20.5%) and retroperitoneum (17%) (4). While less common, ESOS can also arise in the orbits (5), liver (6), kidneys (7), penis (8), breast (9), colon, skin (10), and other anatomical regions. The occurrence of ESOS in the esophagus is exceedingly uncommon, with the first esophageal osteosarcoma case reported by McIntyre et al. (11) in 1982; only three such cases have been documented thus far (11–13). This report detailed the inaugural case of esophageal ESOS with PPM1D-BCAS3 fusion identified post-ESD.

## Case demonstration

A 76-year-old male patient presented with a 2-month history of subxiphoid discomfort and dysphagia. Two months ago, the patient developed subxiphoid pain without obvious inducement, swelling pain and reflux after eating, obstruction feeling when eating, mild cough, expectoration, and white serous sputum. He had a history of hypertension for 3 years. He had a history of smoking and drinking for 50 + years. There was no history of genetic disease or cancer in the family. Physical examination showed T 36.2°C, R 20 beats/min, P 108 beats/min, Bp 160/86 MMHG. The body was thin and the superficial lymph nodes were not palpable. The neck was soft and the trachea was centered. There was no bilateral chest symmetry deformity, bilateral lung breath sounds were clear, and no dry or wet rales were heard.

### Endoscopy

**FIGURE 1:**
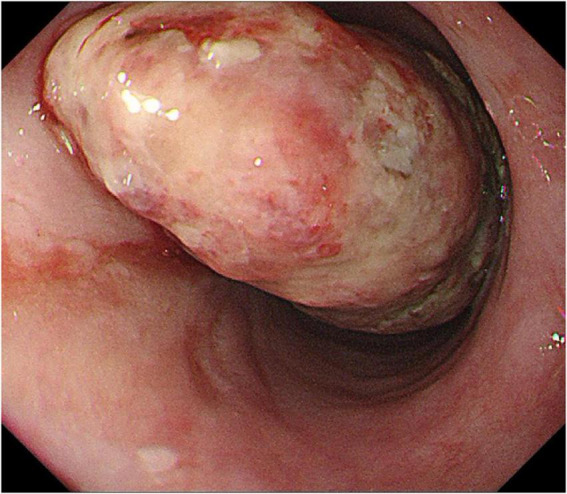
Esophageal endoscopy revealed an intraluminal growing mass in the esophagus 24 to 29 cm from the incisor.

Endoscopic findings: A pedunculated mass was seen protruding into the lumen of the esophagus about 24–30 cm from the incisor tooth, with erosion and bleeding on the surface (Figure 1).

### EUS (endoscopic ultrasonography)

**FIGURE 2:**
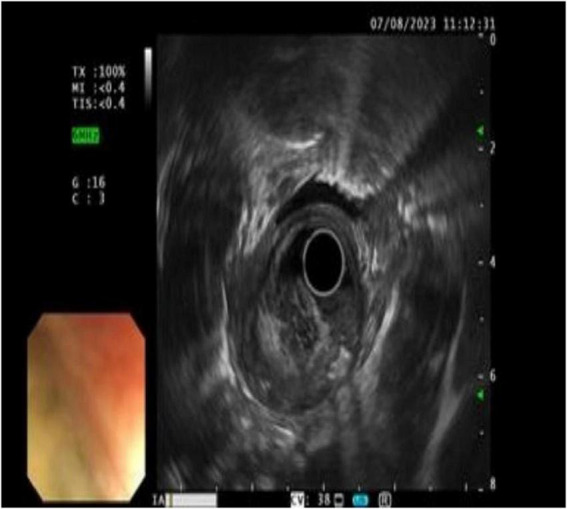
Ultrasound findings: the wall of the esophageal lesions was significantly thickened and hypoechoic, with hyperechoic and fluid anechoic areas in the wall, and some areas had blurred boundaries with the outer membrane.

Ultrasound imaging (Figure 2) revealed pronounced esophageal wall thickening characterized by hypoechoic regions, interspersed hyper- and anechoic fluid areas, and poorly demarcated boundaries between specific segments and the outer membrane. The imaging features were indicative of an esophageal tumor, accompanied by chronic atrophic gastritis with erosive lesions.

### Chest computed tomography (CT)

**FIGURE 3:**
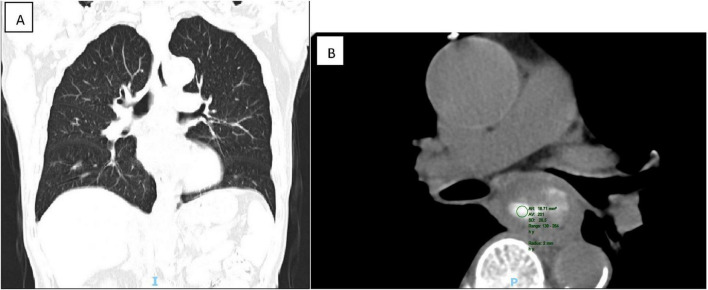
CT showed that the wall of the middle esophagus was uneven and significantly thickened, corresponding to the stenosis of the lumen. The middle esophagus cancer was considered (**A:** coronal view, **B:** transverse view).

Chest computed tomography (CT) identified heterogeneous thickening of the mid-esophageal wall, correlating with luminal stenosis indicative of mid-esophageal carcinoma (Figures 3a,b).

Initial endoscopic biopsy revealed extensive necrosis and exudate on pathology, necessitating further investigation due to the suspicion of malignancy. Consequently, endoscopic submucosal dissection (ESD) was performed to facilitate comprehensive pathological evaluation.

### Gross macroscopic view

The pathological examination revealed a well-demarcated gray-white to gray-brown mass with a smooth surface, measuring 8.0 × 4.5 × 2.5 cm. The cut surface exhibited consistent gray-white to gray-brown coloration, a medium texture, and areas of localized induration.

### Histomorphology

**FIGURE 4:**
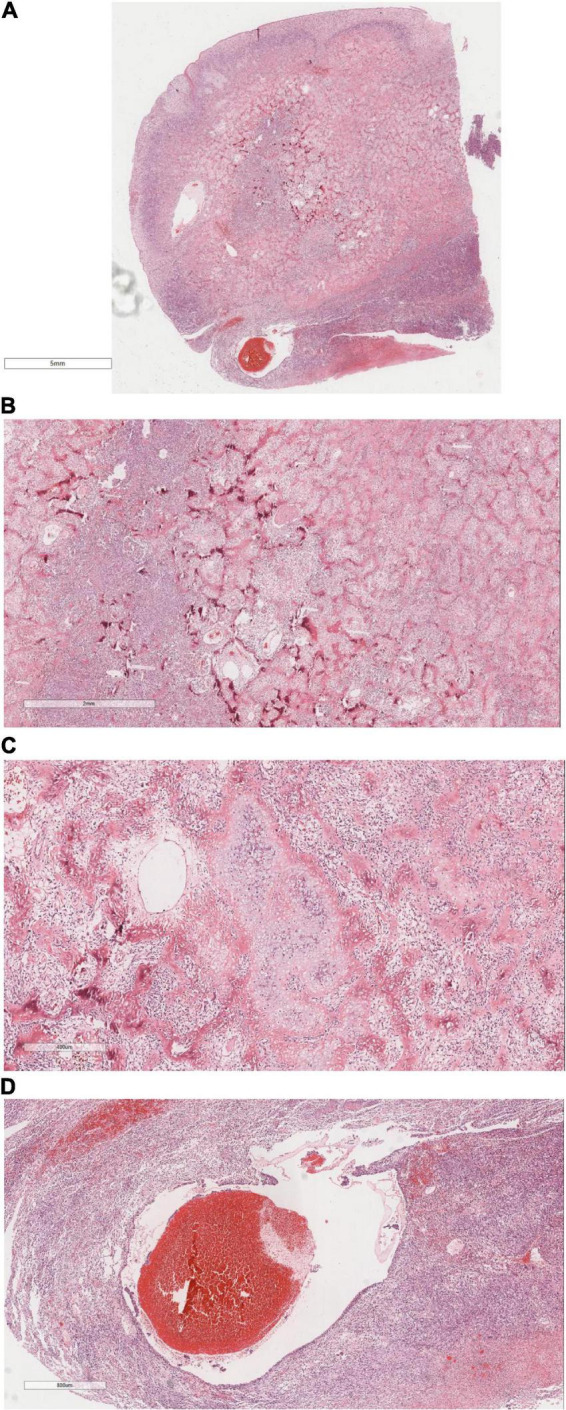
It shows under the microscope, **(A)** The tumor was well circumscribed and covered with a thin layer of esophageal squamous epithelium. H&E × 4. **(B)** Tumor cells were composed of polygonal or rapid-hammer cells, which were nested or braid shaped. H&E × 16. **(C)** The tumor showed focal chondroid differentiation with mild nuclear atypia in the cartilage lacunae. H&E × 50. **(D)** The tumor was hypervascularized. There were large vascular lumens with tumor cells at the edge of the lumens, a large number of red blood cells and eosinophilic homogeneous red staining in the lumens. H&E × 26.

Microscopic analysis (Figures 4A–D) identified a tumor with well-defined borders, encapsulated by a thin esophageal squamous epithelium. The tumor featured densely stained regions comprised of polygonal or fusiform cells organized in nests or interwoven patterns. Cellular atypia was prominent, characterized by vacuolated nuclei, inconspicuous nucleoli, and visible mitotic figures. Focal chondroid differentiation was observed, with markedly atypical nuclei present within the cartilage lacunae. The tumor’s central region exhibited abundant eosinophilic osteoid tissue, forming irregular lace-like and grid-like structures. The vascular network within the tumor was extensive, with large vascular cavities infiltrated by tumor cells, containing numerous erythrocytes and eosinophilic homogeneous red-stained material.

### IHC

**FIGURE 5:**
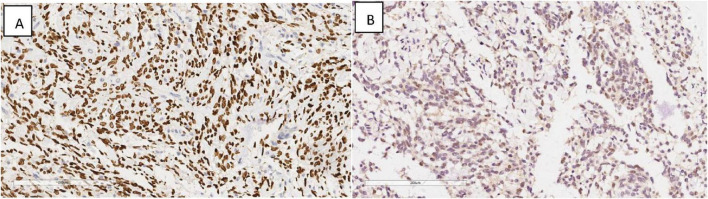
Immunohistochemical results showing tumor cells SATB2 (+) **(A)**, CDK4 (+) **(B)**. EnVision, × 200.

Immunohistochemical profiling demonstrated the presence of Vimentin (+), SATB2 (+) (Figure 5A), CDK4 (+) (Figure 5B), with an absence of CK, CK5/6, P40, Desmin, MDM2, and P16, while SMA showed focal positivity in tumor cells.

### Molecular genetics

**FIGURE 6:**
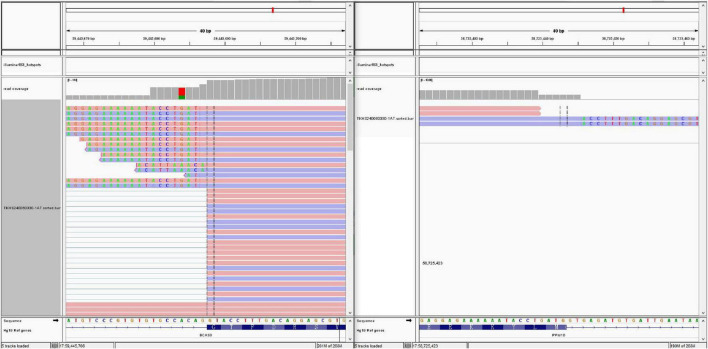
Next-generation sequencing revealed a PPM1D-BCAS3 Fusion P4:B23, with a fusion between exon 4 of the PPM1D gene and exon 23 of the BCAS3 gene.

Targeted next-generation sequencing (Figure 6) identified 1166 gene mutations, notably including a PPM1D-BCAS3 fusion (PPM1D-BCAS3 Fusion P4: B23; the fusion involved exon 4 of PPM1D and exon 23 of BCAS3, with 12 reads confirming the mutation within the sample).

Taking into account the patient’s clinical history, morphological characteristics, immunohistochemical profile, and sequencing data, a pathological diagnosis of mid-esophageal osteosarcoma was rendered. Given the extreme rarity of primary esophageal osteosarcoma, we considered the need to exclude the possibility of carcinosarcoma, so all the samples of the mass were taken and made into HE, but there was no epithelial cancer component in the tumor component, further clinical evaluation is warranted to definitively determine the primary origin and exclude potential metastasis.

### Follow-up

**FIGURE 7:**
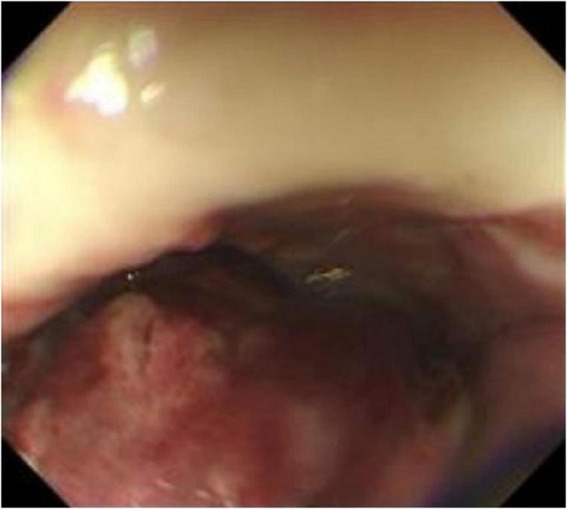
Reexamination of gastroscopy 9 months after surgery showed a large mass in the esophagus 18–30 cm from the incisor, suggesting tumor recurrence.

Nine months after surgery, the patient was readmitted due to postprandial esophageal obstruction. Thin-layer chest CT with 3D reconstruction identified the following: (1) Esophageal carcinoma affecting the middle and upper segments, infiltrating adjacent structures, necessitating further diagnostic assessment; (2) Mediastinal lymphadenopathy indicative of possible metastatic spread. Whole-body skeletal planar imaging combined with fusion CT revealed: (1) Symmetrical hypermetabolic foci in the distal femurs bilaterally, indicative of the “hot patella sign,” with no evidence of osseous lesions or abnormal metabolic activity elsewhere; (2) Subpleural nodules in the right upper lobe and a mediastinal mass demonstrating uptake of 99mTc-MDP. Painless electronic esophagoscopy with NBI detected a large mass within the esophagus, located 18–30 cm from the incisors (Figures 7a,b), consistent with tumor recurrence.

### Diagnosis

The diagnostic findings excluded the possibility of metastatic disease originating from a primary non-esophageal source, thereby confirming recurrent esophageal ESOS with multiple metastases characterized by PPM1D-BCAS3 fusion.

## Discussion

ESOS, a rare mesenchymal malignancy, manifests as an osteosarcoma within extraosseous organs and soft tissues. According to the criteria established by Lee et al. 15, early-stage diagnosis of ESOS necessitates: (1) origin within soft tissues, independent of bone or periosteum; (2) uniform sarcomatous histology, ruling out mixed malignant mesenchymal tumors; (3) osteoid and/or cartilage matrix production. This case aligns with the aforementioned diagnostic parameters.

### Clinical characteristics

In contrast to primary osteosarcoma, which originates in the bones and predominantly affects adolescents, ESOS is more commonly identified in adults over 50 years of age, with a marked male predominance. Prior to this study, only three cases had been reported, making this the fourth documented case. The clinical presentation of these four ESOS cases primarily features dysphagia (Table 1). Onset occurred between ages 67 and 74, with a median age of 72 years. ESOS can manifest in any part of the esophagus. Follow-up periods varied from 1 to 24 months, with three patients succumbing to the disease and one remaining tumor-free. Due to the non-specificity of clinical symptoms, rapid progression, and significant tumor size, ESOS is frequently misdiagnosed as a tumor of esophageal epithelial origin. The etiology remains uncertain, although prior literature suggests potential links to trauma and X-ray exposure (1, 4, 14).

**TABLE 1:** Clinical features of esophageal osteosarcoma.

Case	Document	Sex	Age (year)	Tumor size (cm)	The occurrence of the site	Clinical manifestation	Treatment	Prognosis	Follow-up
1	Deben and Zexin (12)	Male	70	13.0 × 13.0 × 5.0	Lower esophageal segment	No discomfort behind the sternum	Surgical operation	Died of aspiration pneumonia	1
2	Erra et al. (13)	Male	67	7.8 × 4.0 × 2.0	Lower middle and lower esophagus	Progressive dysphagia was associated with retrosternal pain	Surgical operation	There was no recurrence or metastasis	24
3	Erra et al. (11)	Male	74	Maximum diameter 8	Cervical esophagus	Repeated chest pain was associated with dysphagia	Untreated	He died from obstruction and bleeding complications	2
4	This case	Male	76	8.0 × 4.5 × 2.5	Middle esophagus	Subxiphoid pain was associated with feeding obstruction	Endoscopic esophageal mucosal dissection	Tumor recurrence	9

### Imaging manifestations

Large soft tissue masses can be identified through X-rays and CT scans, although imaging alone does not provide definitive differentiation between primary and metastatic lesions. Endoscopic evaluation reveals a solitary, sizable mass with a polypoid morphology. The endoscopic characteristics in this case closely align with those described in cases 1 and 2 in Table 1, suggesting a potentially distinctive presentation of esophageal osteosarcoma. Due to the rarity of reported cases, additional studies involving larger patient cohorts are necessary to validate these findings.

### Pathological characteristics

ESOS typically manifests as a soft tissue mass, predominantly occurring in the thigh, upper limbs, and retroperitoneum, with esophageal involvement being an extreme rarity, documented in only three cases. Tumor sizes vary from 2.5 to 30 cm (15), with this case measuring between 7.8 and 13 cm. ESOS is categorized into six pathological subtypes: osteoblastic, fibroblastic, chondroblastic, giant cell, capillary dilation, and small cell types. Morphologically, it bears a strong resemblance to osteosarcoma, with the osteoblastic subtype being the most common. In this case, extensive osteoid production is observed, characteristic of the osteoblastic subtype, along with the presence of chondroblasts, fibroblasts/pleomorphic malignant fibrous histiocytoma, and capillary dilation. Tumor cells are also noted within the margins of dilated vascular lumina.

The diagnosis of ESOS necessitates ruling out metastatic lesions from other origins before attributing it to a primary tumor. Immunohistochemistry lacks specificity, as it does not express epithelial markers like CK and CK5/6; however, it shows expression of SATB2 and vimentin, with occasional MDM2 positivity. SATB2, a marker highly sensitive to osteoblastic differentiation, is primarily observed in both benign and malignant bone tumors with osteoblastic features, as well as in soft tissue tumors exhibiting heterogeneous bone differentiation (16). In this case, SATB2 and CDK4 expression, along with focal chondrocyte differentiation, were identified. Masson trichrome staining, which highlighted the bone matrix in blue, contributed to the diagnosis (17). Markers such as CK, P40, and P16 effectively excluded common epithelial cell-derived esophageal tumors, while negative staining for SMA, desmin, and MDM2 further supported the differential diagnosis.

### Molecular genetics

Primary osteosarcoma is linked to a spectrum of inherited germline mutations, with TP53 being the most frequently altered gene. Mutations in other significant driver genes, including RB, c-Myc, NOTCH1, FOS, NF2, WIF1, BRCA2, APC, PTCH1, and PRKAR1A, are also prevalent (18). In this case, targeted next-generation sequencing approach identified 1,166 gene mutations and uncovered a PPM1D-BCAS3 fusion.

PPM1D encodes a serine/threonine phosphatase involved in regulating cell cycle arrest. Its overexpression, mutation, and amplification are frequently observed across a spectrum of cancers. PPM1D exerts negative regulation on several tumor suppressor pathways, including ATM, CHK2, p38 MAPK, and p53, contributing to enhanced osteosarcoma proliferation and migration through the upregulation of PKP2, which has potential as an early diagnostic biomarker for the disease (19). Furthermore, TRPM2-AS may exacerbate osteosarcoma malignancy by acting as a molecular sponge for the miR-15b-5p/PPM1D axis (20).

BCAS3 represents a protein coding gene that acts as an acetyltransferase activator, binding to both β-tubulin and histone acetyltransferase. It contributes to cellular responses to estrogen, augments catalytic activity, and promotes RNA polymerase II-driven transcription, while also playing a role in ectoderm differentiation. Evidence suggests BCAS3 mediates the coupling of microtubules with intermediate filaments, thereby supporting cell migration essential for angiogenic remodeling (21). Furthermore, BCAS3, in conjunction with PPM1D, may be implicated in the initiation, progression, and metastasis of osteosarcoma.

### Differential diagnosis

The differential diagnosis for this case included:

1. Metastatic osteosarcoma: This possibility was excluded based on the patient’s medical history and comprehensive imaging, including bone scans, X-rays, and CT, which revealed no primary lesions outside the esophagus.
2. Carcinosarcoma: Consideration was given to carcinosarcoma, a rare biphasic malignancy comprising both squamous cell carcinoma and sarcoma components. The carcinomatous element typically manifests as squamous cell carcinoma, although adenocarcinoma has been occasionally reported. The sarcomatous component usually consists of spindle cells mimicking malignant fibrous histiocytoma, with potential differentiation into muscle, cartilage, or bone. However, the absence of a squamous cell carcinoma component in this case, along with negative epithelial markers CK and CK5/6, ruled out carcinosarcoma.

### Treatment and prognosis

ESOS remains an exceptionally rare malignancy with no established treatment protocol. Current therapeutic strategies focus predominantly on surgical intervention, with the approach determined by the tumor’s anatomical location, size, and extent. Surgical options include local, wide, and radical resections. Although radical resection is preferred for achieving local control, it has no effect on distant metastasis (22). Wide resection offers limited survival advantage. Adjuvant chemotherapy or preoperative radiotherapy may provide additional benefit, particularly in cases of large lesions or positive margins, with chemotherapy typically reserved for palliative purposes. The effectiveness and optimal chemotherapy regimens for ESOS continue to be a subject of debate. Ahmad et al. (23) identified that among 60 ESOS patients (24), received doxorubicin-based chemotherapy, with a response rate of 19%. Wang et al. (25) documented that most patients were administered chemotherapy regimens including methotrexate, doxorubicin, and cisplatin, while a smaller subset received either doxorubicin or ifosfamide. A retrospective analysis by Tsukamoto et al. (26) involving 760 ESOS patients compared outcomes between adjuvant chemotherapy post-local resection and post-surgical resection. The 5-year disease-free survival rate was 47.9% (187/390) in the surgery plus (neo)adjuvant chemotherapy cohort, versus 40.4% (150/371) in the surgery-only cohort. The addition of (neo)adjuvant chemotherapy did not significantly enhance the 5-year disease-free survival rate, indicating its limited efficacy for localized ESOS. Chemotherapy regimens for advanced and metastatic ESOS have consistently resulted in poor outcomes. Although some studies suggest radiotherapy or systemic chemotherapy as treatment options, an established standard of care remains elusive. In Torigoe et al. (24), a partial response was observed in 5 out of 11 patients treated with doxorubicin and cisplatin, yielding a 45% response rate. Despite the utilization of various chemotherapy regimens for advanced and metastatic disease, therapeutic outcomes remain inadequate. Although radiotherapy and systemic chemotherapy have been applied in certain instances, an established standard of care for ESOS remains elusive. ESOS is characterized by aggressive behavior, with a high propensity for local recurrence and metastasis.

In this case, the tumor recurred 9 months postoperatively, followed by metastasis to the lungs and mediastinal lymph nodes. Tumor size may play a critical role in determining prognosis. Further research is necessary to clarify the relationship between histological subtype, clinicopathological features, and patient outcomes. This patient did not undergo lymph node dissection, the absence of pre-ESD EUS staging limits the rationale for ESD, as initial tumor depth and lymph node status were not evaluated, so there is a certain limitation in the evaluation of tumor staging and a certain reference basis for guiding the subsequent treatment.

## Conclusion

ESOS, an aggressive malignancy, typically originates in the deep soft tissues of the lower and upper extremities, as well as the retroperitoneum, with esophageal involvement being exceptionally uncommon. Accurate diagnosis demands a thorough approach, incorporating clinical history, imaging, and histopathology to differentiate it from metastatic osteosarcoma and carcinosarcoma. Due to the poor prognosis associated with ESOS, early detection and surgical intervention are imperative. This report details the first documented case of esophageal ESOS with PPM1D-BCAS3 fusion treated via ESD, contributing valuable insights to clinical and pathological practice. The management of ESOS necessitates vigilant monitoring and diligent follow-up care.

## Data Availability

The original contributions presented in this study are included in this article/supplementary material, further inquiries can be directed to the corresponding author.
